# Warrior Patients in Hospitals: Their Temperatures and Maladies

**Published:** 1917-05-26

**Authors:** 


*To the Editor of* THE HOSPITAL.

### Warrior Patients in Hospitals: their Temperatures and Maladies.

*To the Editor of* THE HOSPITAL.

SIR,—I shall be glad if you will give me your opinion on the following matter. I am matron of a small V.A.D. hospital, and have had wide previous experience in hospital work. In every hospital in which I have worked it has been the invariable custom to hang the temperature chart over the patient's bed, with a brief memorandum of the nature of the malady. This practice I have carried out during the last year in my present hospital. To my astonishment the military medical inspector at his last visit took exception to this, and expressed annoyance that the men should be permitted to know what was the matter with them, and whether or no they "had a temperature." When a man has had part of his arm shot away it seems rather a counsel of perfection to attempt to conceal from him the nature of his malady, but in any case I should be very glad, Sir, if you could inform me whether it is the practice now in the best military hospitals to keep the charts hidden from the men. I may add that I have not been able to meet any nurse who is aware of any other custom but that of hanging the chart over the head of the bed. PERPLEXED.

[There are cases in which it is desirable that the patient should not know whether his temperature is rising or falling; this holds good of civilian as well as of military patients. If a medical officer suddenly gives orders that a particular patient's chart is to be concealed from his gaze (and that of the other patients in the ward), the individual affected may immediately conclude that his case is desperate and his courage may fail him just when it is important that he should keep a stout heart. The detection of malingering is also facilitated if the suspected individual has no idea what his temperature is. For these and other reasons of the same sort it is comprehensible that a hospital administrator might order the concealment of charts. As a matter of actual fact the practice in military hospitals varies: we know of some where the patients never know what their temperatures are, and of others where they always do so. The matter is one for the discretion of the individual medical officer, who in his turn may be subject to the decrees of his military superior, the commanding officer.—ED. THE HOSPITAL.]

(*Continued on page* 160.)

